# Influenza Virus A (H10N7) in Chickens and Poultry Abattoir Workers, Australia

**DOI:** 10.3201/eid1805.111852

**Published:** 2012-05

**Authors:** George G. Arzey, Peter D. Kirkland, K. Edla Arzey, Melinda Frost, Patrick Maywood, Stephen Conaty, Aeron C. Hurt, Yi-Mo Deng, Pina Iannello, Ian Barr, Dominic E. Dwyer, Mala Ratnamohan, Kenneth McPhie, Paul Selleck

**Affiliations:** Elizabeth Macarthur Agricultural Institute, Menangle, New South Wales, Australia (G.G. Arzey, P.D. Kirkland, K.E. Arzey, M. Frost);; Sydney South West Public Health Unit, Sydney, New South Wales, Australia (P. Maywood, S. Conaty);; World Health Organization Collaborating Centre for Reference and Research on Influenza, North Melbourne, Victoria, Australia (A.C. Hurt, Y.-M. Deng, P. Iannello, I. Barr);; Westmead Hospital Centre for Infectious Diseases and Microbiology, Westmead, New South Wales, Australia (D.E. Dwyer, M. Ratnamohan, K. McPhie);; Commonwealth Scientific and Industrial Research Organisation Australian Animal Health Laboratory, East Geelong, Victoria, Australia (P. Selleck)

**Keywords:** Influenza A, influenza A (H10N7), chickens, humans, H10N7, influenza, respiratory infections, viruses, poultry abattoir, workers, Australia, poultry, New South Wales, *Suggested citation for this article*: Arzey GG, Kirkland PD, Arzey KE, Frost M, Maywood P, Conaty S, et al. Influenza virus A (H10N7) in chickens and poultry abattoir workers, Australia. Emerg Infect Dis [serial on the internet]. 2012 May [*date cited*]. http://dx.doi.org/10.3201/eid1805.111852

## Abstract

In March 2010, an outbreak of low pathogenicity avian influenza A (H10N7) occurred on a chicken farm in Australia. After processing clinically normal birds from the farm, 7 abattoir workers reported conjunctivitis and minor upper respiratory tract symptoms. Influenza virus A subtype H10 infection was detected in 2 workers.

Reported outbreaks of low pathogenicity avian influenza (LPAI) viruses of influenza A subtype H10 in poultry are uncommon but have occurred among turkeys and emus in the United States (*1**,**2*), farmed Pekin ducks in South Africa (*3*), and chickens in Canada (*4*). Isolation of influenza virus A (H10N7) was reported in Italy from smuggled poultry products from China (*5*). Transmission of LPAI viruses from birds to humans, resulting in symptomatic disease, has been reported for influenza virus A subtypes H9N2 in China and Hong Kong, H7N2 in North America and the United Kingdom, H7N3 in Canada, H7N7 in the United Kingdom (*6*), and H10N7 in Egypt (*7*).

## The Study

In March 2010, an outbreak of LPAI A (H10N7) was identified in a biosecure intensive commercial poultry enterprise in New South Wales, Australia. For 8–14 days, 10–25 birds died each day, compared with the normal number of 2–6 birds per day. An egg production decrease of up to 15% was documented in the affected flocks. In contrast to other reported poultry outbreaks (*1**–**4*), respiratory signs were absent in the flock.

All cloacal and tracheal swabs from 10 dead and 10 live birds submitted for influenza virus exclusion were positive by an influenza A matrix gene quantitative real-time reverse transcription PCR (*8*), and virus was detected at various levels (cycle threshold [C_t_] 15–37). The influenza A viruses were then subtyped from swabs by using a microarray assay (Clondiag, Jena, Germany) that enabled the rapid identification of influenza virus A (H10N7). The virus was readily cultured from swabs in embryonated chicken eggs and in MDCK cell cultures. Several viral genome segments were sequenced, which enabled confirmation of the virus as LPAI A (H10N7) and performance of phylogenetic analysis. A fluorescence-based neuraminidase inhibition assay showed the isolate to be sensitive to the antiviral drugs oseltamivir and zanamivir (mean 50% inhibitory concentration ± SD 0.5 ± 0.1 nmol/L and 1.8 ± 0.3 nmol/L, respectively) (*9*).

Serologic testing was conducted by using an influenza A nucleoprotein–based blocking ELISA and a subtype H10–specific hemagglutination inhibition test; results showed widespread infection in the affected flock, with 18 of 20 samples seropositive. Sampling across a 4 additional flocks on site showed that an additional 9 of 40 birds were seropositive for influenza A subtype H10.

Ten days after the outbreak was confirmed, 3 previously seronegative flocks from the site were sent to an abattoir; 1 day earlier, they had passed state government clinical inspection, including inspection and examination of production and mortality records. Within a week, 7 workers at the abattoir showed signs of conjunctivitis; 2 also reported rhinorrhea and 1 a sore throat.

Conjunctival swabs were collected from 6 of the workers and nose and throat swabs from all 7. Influenza A RNA was detected by PCR 4 days after abattoir exposure in conjunctival swabs from a worker who reported conjunctivitis, rhinorrhea, and sore throat (C_t_ 31.8) and 7 days after abattoir exposure from the nose/throat swab of another worker who reported only conjunctivitis with onset 2 days earlier (C_t_ 35). Partial sequence analysis of the hemagglutinin genes from both samples (GenBank accession nos. CY063325 and CY063326) confirmed the presence of influenza A subtype H10; the partial sequences were identical to the subtype H10 chicken isolate, although no virus was cultured from the workers.

The conjunctivitis and other reported symptoms among the 7 workers were mild and of short duration, and there was no evidence of seroconversion by hemagglutination inhibition or virus neutralization tests in any of the workers from whom convalescent-phase blood was collected, including the 2 with confirmed influenza A subtype H10 infection. These findings are consistent with the mild symptoms and lack of serologic evidence reported in humans after experimental infection with influenza A (H10N7), which may indicate the limited ability of the virus to multiply and stimulate a detectable immune response in humans (*10*). Other studies have reported no evidence of elevated subtype H10–specific antibody titers among poultry abattoir workers, although serologic evidence of subtype H10 infection was detected among turkey farmers in the absence of clinical symptoms (*11*).

Although 4 farm staff members from the site of the initial infections reported conjunctivitis and other symptoms to health care workers, influenza was not confirmed. The abattoir workers with laboratory-confirmed influenza A subtype H10 infection handled offal and giblets in a section of the abattoir where automated evisceration usually took place; however, because of the size of the birds, evisceration was manually assisted on the day that these flocks were slaughtered.

No obvious breach of biosecurity occurred on the farm. The water supply to the farm was chlorinated town water; no large dams were on site, only small paddock dams for cattle. The sheds were birdproof and protected by additional bird netting. A feed mill supplied the feed, which was delivered into silos through blow pipes from outside the perimeter fence. Litter (wood shavings) was delivered in enclosed bales. Workers showered on the way in and out of facilities; disinfectant foot baths were placed at the entrance of each shed, and staff were required to use the separate footwear provided inside the shed. Staff were not allowed to have birds or pigs at home.

## Conclusions

During 2010, the number of wild waterfowl observed on the affected site was unusually low. Surveillance of poultry flocks within a 2-km radius of the affected farm did not detect any serologic or virologic evidence of subtype H10 infection. Ongoing surveillance of wild waterfowl in New South Wales reported influenza virus A (H10N7) in other areas in the previous year (K.E. Arzey, unpub. data); however, during 2007–2008, onsite surveillance detected no evidence of influenza A infection among wild waterfowl (G.G. Arzey, unpub. data).

The phylogenetic analysis of the full hemagglutinin sequence from the influenza A (H10N7) infections reported here in chickens showed a high degree of homology with North American avian influenza A subtype H10 viruses (Figure). This is an unusual finding, given that most avian influenza viruses detected in Australia are related to Eurasian avian influenza strains (*12*).

**Figure:**
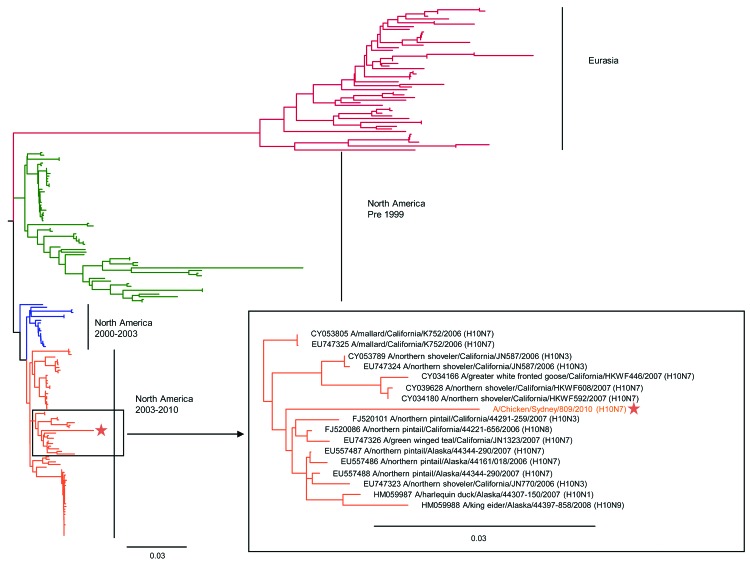
Phylogenetic analysis of avian influenza subtype H10 hemagglutinin (HA) sequences. HA sequences of all subtype H10 viruses deposited in GenBank were downloaded, and a neighbor-joining tree was created by using Jukes-Cantor as the genetic distance model on Geneious 5.14 software (Biomatters Ltd, Auckland, New Zealand) and a phylogenetic tree drawn by using FigTree version 1.3.1 (http://tree.bio.ed.ac.uk/software/figtree/). A representative HA sequence from the subtype H10N7 viruses detected from the New South Wales chicken farm outbreak (A/chicken/Sydney/809/2010) has been submitted to the Global Initiative on Sharing All Influenza Data (accession no. EPI339225) and is marked by the star in the tree. Scale bar indicates nucleotide substitutions per site.

The finding of LPAI in commercial poultry in Australia is rare, with only 2 reports published (*13*). To our knowledge, no transmission to humans has been reported previously, including during the 5 reported HPAI outbreaks in Australia during 1976–1997 (*13*). The United Kingdom Advisory Committee on Dangerous Pathogens (*14*) acknowledged the occupational risk from slaughter of LPAI-infected birds and recommended that appropriate personal protective equipment (PPE) be used during handling of infected birds. However, because of the recent testing of the flock, the birds in this instance were assumed to be free of infection, and workers did not wear goggles and face masks in the evisceration section on the day the flocks were processed. After this incident, use of full PPE, including the use of enclosed rim goggles and P2 (N95) face masks, was implemented in sections of the abattoir where exposure to birds and carcasses from the infected site was likely. Staff training, compliance with PPE use, and the decision to slaughter flocks from the site only after a sufficient number of birds were sampled to enable detection of a low infection prevalence and at a 99% confidence level resulted in no further reported infection of workers during the processing of all flocks from the site over 10 months. Analysis of preslaughter samples showed that the spread of infection stopped about 4 weeks after influenza virus A (H10N7) was first confirmed on the site.
